# Self-perception of nurses’ competence in family assessment and intervention

**DOI:** 10.17533/udea.iee.v39n3e13

**Published:** 2021-11-08

**Authors:** Maria Henriqueta Figueiredo, Maria Manuela Ferreira, Marlene Lebreiro da Silva, Virgínia Sousa Guedes

**Affiliations:** 1 Nurse, Ph.D. Professor, Coordinator of Escola Superior de Enfermagem do Porto, Portugal. Email: henriqueta@esenf.pt. Corresponding author Escola Superior de Enfermagem do Porto Escola Superior de Enfermagem do Porto Portugal henriqueta@esenf.pt; 2 Nurse, Ph.D. Adjunct Professor at Escola Superior de Saúde Norte da Cruz Vermelha Portuguesa. Email: manuela.ferreira@ essnortecvp.pt Escola Superior de Saúde da Cruz Vermelha Portuguesa Escola Superior de Saúde Norte da Cruz Vermelha Portuguesa Portugal manuela.ferreira@ essnortecvp.pt; 3 Family Nurse. Family Nurse at grupamento de Centros de Saúde Porto Ocidental, Portugal. Email: enfmarlenelebreiro@gmail.com Centros de Saúde Porto Ocidental Portugal enfmarlenelebreiro@gmail.com; 4 Nurse, Master's. Family Nurse at Agrupamento de Centros de Saúde Tâmega I - Baixo-Tâmega, Portugal. Centros de Saúde Tâmega I Baixo-Tâmega Portugal

**Keywords:** primary health care, nursing process, family nursing., atención primaria de salud, proceso de enfermería, enfermería de la familia., atenção primária à saúde, processo de enfermagem, enfermagem familiar.

## Abstract

**Objective:**

To describe nurses’ self-perception of competence in family assessment and intervention

**Methods:**

A sample of 551 Portuguese primary care nurses was selected. A Likert-type questionnaire with 11 items corresponding to the areas of care proposed by the Dynamic Model of Family Assessment and Intervention (MDAIF) was administered. Each item consists of 7 optional responses; a score equal to or greater than 4 denotes competence.

**Results:**

The nurses perceived themselves as competent in areas of care belonging to the development dimension of the MDAIF (parental role, adaptation to pregnancy, and family planning), as well as in the caregiver role (which belongs to the functional dimension). There was a progressive decline in self-perception of competence over the stages of the nursing process.

**Conclusion:**

In this study, crucial aspects related to nurses’ self-perception of their competence in family assessment and intervention were observed, and need to be addressed in the training of nurses in all areas of care included in the Model. This should facilitate awareness of the competences needed to provide the best care for families.

## Resumen

**Objetivo:**

Describir la autopercepción de la competencia del enfermero en la valoración e intervención familiar.

**Métodos:**

Estudio exploratorio descriptivo de naturaleza cualitativa. Se seleccionó una muestra de 551 enfermeros portugueses de atención primaria. Se utilizó un cuestionario con opciones de respuesta tipo Likert, con 11 ítems correspondientes a las áreas de atención propuestas por el Modelo Dinámico de Evaluación e Intervención Familiar. Cada ítem tiene 7 opciones de respuesta, y se considera competente un valor igual o superior a 4.

**Resultados:**

Los enfermeros se autoperciben como competentes en las áreas de atención de la dimensión de desarrollo del Modelo (rol parental, adaptación al embarazo y planificación familiar), así como en el rol de brindar cuidados, en el que se realza que esta última área está integrada en la dimensión funcional. Se destaca también la disminución progresiva de la autopercepción a lo largo de las etapas del proceso de enfermería.

**Conclusión:**

En este estudio se observó que hay aspectos cruciales relativos a la autopercepción de la competencia de los enfermeros en la evaluación e intervención familiar que necesitan ser intervenidos en la formación de estos profesionales en todas las áreas de cuidados del Modelo, lo que facilitará la concienciación de las habilidades necesarias para proporcionar los mejores cuidados a las familias.

## Resumo

**Objetivo:**

Descrever a autopercepção da competência do enfermeiro na avaliação e intervenção familiar.

**Métodos:**

Foi selecionada uma amostra de 551 enfermeiros portugueses dos cuidados de saúde primários. Foi utilizado um questionário com opções de resposta tipo Likert, com 11 itens correspondentes às áreas de atenção propostas pelo Modelo Dinâmico de Avaliação e Intervenção Familiar. Cada item integra 7 respostas opcionais, sendo considerado competente um valor igual ou superior a 4.

**Resultados:**

Os enfermeiros autopercionaram-se como competentes, em áreas de atenção da dimensão de desenvolvimento do MDAIF (papel parental, adaptação à gravidez e planeamento familiar), assim como no papel de prestador de cuidados, realçando-se que esta última área está integrada na dimensão funcional, destacando também uma progressiva diminuição da autopercepção ao longo das etapas do processo de enfermagem.

**Conclusão:**

Neste estudo observou-se que existem aspectos cruciais relacionados à autopercepção da competência do enfermeiro na avaliação e intervenção familiar que precisam ser tratados na formação do enfermeiro em todas as áreas de cuidado do Modelo, facilitando a conscientização de competências necessário para proporcionar o melhor cuidado às famílias.

## Introduction

Primary health care (PHC) focuses on health promotion and protection and on preventive activities designed to identify risk factors for health. Health surveillance, management and care planning in the health system aim to empower individuals, families, or communities with regard to lifestyle decisions.(1) The main objective of PHC reforms is to strengthen the health system, given that its quality determines the health of populations. From this perspective, health policies aim to obtain the best outcomes by integrating quality improvement in the provision of care as well as increasing effectiveness, so that PHC can provide effective responses to the population’s needs.(2,3) the growing requirement for PHC is closely associated with demographic changes, as well as with greater diversity of family arrangements and interactions, leading to a need to adapt this level of care to the new profile of changing populations.(4) Special emphasis is given to changes in family structure related to the last stage of the family life course, characterized by aging-related transitions.

The prevailing concept of care influences the quality of the provision of nursing care, enabling acquisition of practical skills based on the assimilation and mastery of nursing core content. Theoretical models also provide a foundation from which knowledge of the field of nursing can be both observed and developed.(5) The Dynamic Model of Family Assessment and Intervention (MDAIF) is a theoretical-methodological framework for the provision of family-centered nursing care. It was developed through action research on the practices of PHC family nurses,(6) and was designed to add to the knowledge base of family nursing and inform the clinical practice of family nurses. Implementation of this framework has been perceived by nurses as essential to improve the quality of clinical practice.(7) Its assumptions and principles focus on the resources and strengths of families through a collaborative approach that seeks to empower families.(8)

The MDAIF consists of an operational matrix, with diagnoses and interventions linked to the following dimensions: structural (family income; residential building; safety precaution; water supply; household pets); developmental (marital satisfactions; family planning; adaptation to pregnancy; parental role) and functional (caregiver role; family process). This matrix describes the areas of family assessment and intervention according to the nursing process methodology: family assessment, diagnosis, planning of interventions, and evaluation of the results of implementation. The nursing process methodology can be understood as the process of articulating between management and care provision in order to respond to the client’s needs.(9) It is a systematic technique for data collection, definition of diagnoses, planning, implementation, and evaluation of the care provided. The effectiveness of interventions can be compromised when implementation of the nursing process is inadequate.(10)

The provision of care centered on families’ needs entails that nurses be competent in deploying nursing process methodology, as well as in assimilating the assumptions and clinical guidelines of nursing models. The implementation of competence acquisition and development strategies will contribute greatly to the continuous improvement of the provision of nursing care, considering the concept of competence as the ability to integrate resources and knowledge and mobilize these elements for effective action, leading to an active restructuring of knowledge.(11) If the perception of personal competence occurs in relationship with work, “perception” refers to the assessments people make based on their social representations, associated with the circulation and transformation of ideas in society, allowing the preservation of a rewarding personal and social identity.(6)

The aim of this study is to describe nurses' self-perception of competences in family assessment and intervention, considering the stages of the nursing process. The results of this study seek to contribute to identification of the most common practices of nurses when care becomes family-centered, as well as of self-perception of competence in key areas for the provision of care. These results are certain to also enable identification of training needs and thus lead to an improvement in the quality standards of nursing care provided in the context of PHC.

## Methods

Study setting and population. This is an exploratory, descriptive, quantitative study*.* The participants were nurses working in integrated primary health units in Portugal. The sample included 551 nurses working in PHC in the North of Portugal. The sample was non-probabilistic, and considered the following inclusion criteria: 1) nurses without postgraduate training in family health nursing; and 2) nurses who applied to the MDAIF Framework for Decision Making in Family Nursing course.

Instruments and data collection. Data were collected through a questionnaire based on the operational matrix of the MDAIF, namely with regard to areas of care which cut across all stages of the nursing process. Content validity was attested through expert consensus and pre-testing. No changes were required after this stage, confirming the clarity and relevance of the original items. This instrument was developed in 2012 with a sample of 161 nurses working in Portugal, recruited with the same inclusion criteria.(7) The questionnaire includes items designed to collect sociodemographic and professional data. It includes questions related to assessment of the respondent’s self-perception of competence regarding family assessment and intervention at each stages of the nursing process, based on the areas of care defined for the operational matrix of the MDAIF.(6,13) A Likert-type scale was used for this part of the questionnaire, with 7 response choices, anchored at 1 (“totally incompetent”), 4 (“competent”), and 7 (“totally competent”). Each of the 11 items corresponded to an area of care defined in the MDAIF, conceptualized in its components and operationalized in its operational matrix, taking into account all stages of the nursing process.(6) The questionnaire was self-administered, and data collection was carried out during the year 2020.

Statistical analysis. Statistical analyses were performed in SPSS Version 23.0 for Windows (SPSS Inc., Chicago, IL, USA). Categorical variables were described as absolute and relative frequencies, and continuous variables, as mean and standard deviation (SD) or median and interquartile range (IQR = P75-P25) as appropriate. To determine the total self-perceived competence for each stage of the nursing process, the sum of the items corresponding to each stage of the nursing process was independently calculated and divided by 11 (number of items corresponding to each step of the evaluated nursing process).

Ethical considerations. All ethical considerations and principles were followed and anonymity was ensured, and participation was entirely voluntary and informed. Participants were asked to sign an informed consent form and were informed that they could withdraw from the study at any time during their participation. Participants received all necessary information about the study before enrollment, and were given the opportunity to elucidate any doubts. The study was part of a research protocol between the institution of higher learning where the MDAIF project was developed and the health facilities where the participants work. Once the relevant ethics committee had approved the conduction of the study, institutional authorization was formalized in document No. 217/2019.

## Results

Sample profile. The sample comprised 551 nurses. Most participants were female (89.5%), aged between 23 and 61 years, with a mean age of 39.27 years (SD = 7.23 years). Regarding academic background, the majority of participants had a bachelor's degree in nursing (n = 478, 86.4%); 60 had a master’s degree (10.8%) and 44 held a doctorate (0.7%). There was great heterogeneity in the sample regarding the overall duration of nursing practice, which ranged from 1 year to 39 years, with an average of 15.8 years (SD = 7.28 years). The average number of years of professional practice in PHC specifically was 10.8 years (SD = 7.1 years), ranging from less than 1 year to 37 years.

Self-perception of competence in family assessment and intervention at each stage of the nursing process:

### Needs assessment and diagnostic formulation

In relation to most items, participants perceived themselves as competent, whether in identifying needs or in stating the diagnosis, with mean values close to 4 on the Likert scale. According to Table 1, the lowest average value was found for family income, followed by marital satisfaction and water supply.

Regarding self-perception of competence in nursing diagnosis, the items with an average value of less than 4 were, in ascending order: family income, household pets, residential building, water supply, marital satisfaction, family process, and safety precaution. At this stage, safety precaution and family process presented average values close to 4, but lower than those obtained in the previous stage of the nursing process (needs assessment).

**Table 1:** Self-perception of competence in family assessment and intervention at two stages of the nursing process: needs assessment and nursing diagnosis

Items	Needs assessment	Needs assessment	Needs assessment	Nursing diagnosis	Nursing diagnosis	Nursing diagnosis
Items	*n*	Mean (SD)	Median (IQR)	*n*	Mean (SD)	Median (IQR)
Item 1 Family income	551	3.52 (1.37)	4 (1)	549	3.34 (1.29)	4 (2)
Item 2 Residential building	550	3.86 (1.36)	4 (1)	549	3.55 (1.31)	4 (1)
Item 3 Safety precaution	551	4.05 (1.24)	4 (1)	550	3.83 (1.29)	4 (1)
Item 4 Water supply	549	3.89 (1.55)	4 (2)	550	3.55 (1.48)	4 (1)
Item 5 Household pets	551	3.85 (1.50)	4 (1)	549	3.53 (1.37)	4 (1)
Item 6 Marital satisfaction	551	3.79 (1.36)	4 (1)	550	3.67 (1.33)	4 (1)
Item 7 Family planning	549	4.35 (1.21)	4 (1)	549	4.29 (1.31)	4 (1)
Item 8 Adaptation to pregnancy	551	4.54 (1.30)	4 (2)	549	4.29 (1.30)	4 (1)
Item 9 Parental role	551	4.53 (1.27)	4 (1)	549	4.27 (1.29)	4 (1)
Item 10 Caregiver role	550	4.64 (1.23)	4 (2)	549	4.36 (1.25)	4 (1)
Item 11 Family process	550	4.02 (1.34)	4 (2)	548	3.82 (1.31)	4 (1)
Total score	545	4.08 (1.03)	4 (1)	542	3.85 (1.07)	4 (1)

### Planning, implementation and evaluation of interventions

The items that showed the highest mean score for self-perceived competence in planning interventions (Table 2) were family planning and adaptation to pregnancy. All other items had an average score of less than 4, with the lowest average value for family income. At the implementation of interventions stage of the nursing process (Table 2), the best self-perception of competence was observed for the same items as in each of the previous stages of the nursing process-in ascending order, with an average score greater than 4: parental role, adaptation to pregnancy, family planning, and caregiver role. 

At the evaluation of interventions stage (Table 2), the items with the best self-perceived competence scores were again the same as in each of the previous stages, presented in ascending order according to the degree of agreement with self-competence (average score greater than 4): parental role, adaptation to pregnancy, family planning, and caregiver role. Items at the evaluation of interventions stage were also organized in descending order according to the degree of self-perceived competence of implementation: family process, safety precautions and marital satisfaction, household pets, residential building, water supply and, finally, with the lowest average self-perception score, family income.

**Table 2:** Self-perception of competence in family assessment and intervention at three stages of the nursing process: planning, implementation and evaluation of interventions

Items	Planning of	Planning of	Planning of	Implementation of interventions	Implementation of interventions	Implementation of interventions	Evaluation of interventions	Evaluation of interventions	Evaluation of interventions
Items	*n*	Mean (SD)	Median (IQR)	*n*	Mean (SD)	Median (IQR)	*n*	Mean (SD)	Median (IQR)
Item 1 Family income	550	3.28 (1.35)	3 (2)	549	3.25 (1.40)	3 (2)	520	3.23 (1.42)	3 (2)
Item 2 Residential building	549	3.34 (1.36)	3 (2)	549	3.32 (1.41)	3 (2)	521	3.30 (1.41)	3 (2)
Item 3 Safety precaution	547	3.74 (1.31)	4 (1)	549	3.75 (1.34)	4 (1)	519	3.62 (1.38)	4 (1)
Item 4 Water supply	550	3.37 (1.41)	4 (2)	549	3.31 (1.44)	3 (2)	520	3.31 (1.46)	3 (2)
Item 5 Household pets	550	3.47 (1.35)	4 (1)	547	3.49 (1.37)	4 (1)	520	3.40 (1.42)	4 (2)
Item 6 Marital satisfaction	549	3.66 (1.31)	4 (1)	547	3.69 (1.34)	4 (1)	520	3.62 (1.41)	4 (1)
Item 7 Family planning	549	4.30 (1.27)	4 (1)	548	4.27 (1.28)	4 (1)	520	4.15 (1.36)	4 (2)
Item 8 Adaptation to pregnancy	549	4.31 (1.24)	4 (1)	548	4.26 (1.27)	4 (1)	520	4.14 (1.34)	4 (2)
Item 9 Parental role	550	4.31 (1.25)	4 (1)	548	4.23 (1.25)	4 (1)	520	4.12 (1.37)	4 (2)
Item 10 Caregiver role	548	4.41 (1.23)	4 (1)	548	4.40 (1.24)	4 (1)	520	4.25 (1.32)	4 (1)
Item 11 Family process	549	3.89 (1.34)	4 (2)	548	3.86 (1.33)	4 (2)	520	3.76 (1.39)	4 (1)
Total score	543	3.83 (1.03)	4 (1)	543	3.79 (1.05)	4 (1)	506	3.72 (1.61)	4 (2)

**Figure 1:**
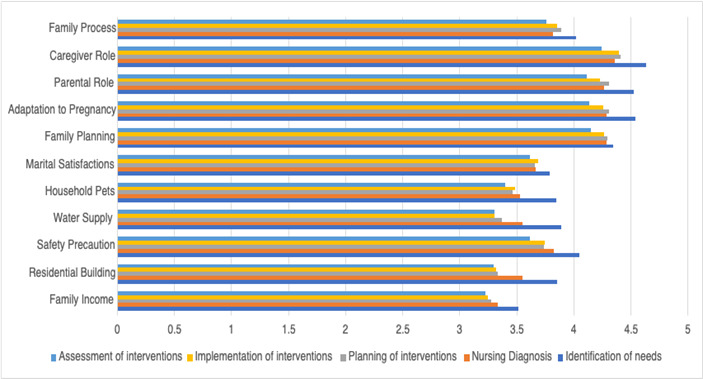
Distribution of mean scores for self-perceived competence in family assessment and intervention at each stage of the nursing process

Figure 1 illustrates the mean distribution of participants' self-perception of competence in family assessment and intervention at all stages of the nursing process: needs assessment, diagnosis, planning of interventions, implementation of interventions, and evaluation of interventions.

The items that corresponded to the care areas family planning, adaptation to pregnancy, parental role, and caregiver role had the highest mean self-perception of competence scores; this trend persisted across the different stages of the nursing process.

At all stages of the nursing process, the caregiver role item had the highest mean self-perception of competence scores, higher than for any other items. This result expresses a self-perception of competence by the participants which exceeds the cutoff score of 4 (competent), on a continuum ranging from 1 (totally incompetent) to 7 (totally competent). Thus, we can conclude that, although the caregiver role item had the highest mean values of self-perceived competence, these scores were still low and far from the maximum possible value.

As shown in Tables 1 and 2 and Figure 1, the areas of care family income, residential building, safety precautions (except at the needs assessment stage), water supply, household pets, marital satisfaction and family process (except at the needs assessment stage) had the lowest mean scores, across all stages of the nursing process. We can also conclude that the participants perceived themselves as incompetent for some items, with mean scores ranging from 3.23 (family income item at the evaluation of interventions stage) to 3.89 (water supply item at the needs assessment stage), on a scale of 1 (totally incompetent) to 7 (totally competent).

### Total scores for all stages of the nursing process

To gain a better understanding of the phenomenon of interest, total mean scores (the sum of all items that correspond to each of the MDAIF care areas) were calculated for each stage of the nursing process. This procedure showed that the average overall score of self-perceived competence decreases as the nursing process progresses (Table 1 and 2). At the needs assessment stage, average self-perception scores were 4.08, declining to 3.85 at the diagnostic formulation stage, 3.83 at the planning of interventions stage, 3.79 at implementation of interventions and, finally, 3.72 at evaluation of interventions.

## Discussion

Descriptive analysis of the participating nurses' self-perception of competence showed that the highest mean item score at each stage of the nursing process was 4.64, in the care area “caregiver role” and at the needs assessment stage, which is clearly a low level. These results are in line with those of a previous study,(14) in which the highest mean score was 4.60, also in the same area of care and stage of the nursing process. At all stages of the nursing process, the care areas caregiver role, parental role, adaptation to pregnancy, and family planning showed average scores between 4.23 and 4.64. These results suggest that these are the areas in which nurses feel most experienced in the context of interacting with families.

The nurse's competence will influence responses related to fertility regulation and preparation for parenthood, as couples have the right to information, as well as access to effective contraceptive methods, promoting reproductive health.(15,16) Regarding adaptation to pregnancy, the nurse's competence will allow identification of the resources of family systems, establish action plans for the couple(17,18) and improve perception of self-efficacy, especially in unwanted pregnancies.(19) The parental role affects other domains of the family, especially when dysfunctional problems arise. This can reduce the time available for the couple, affect child development, decrease interactions with extended family members and affect the social life of all relatives.(20) The caregiver role had higher mean scores than any other area of care, with a minimum of 4.25 (at the evaluation of interventions stage) and a maximum of 4.64 (at the needs assessment stage). The support that nurses provide to the families and caregivers of a dependent patient explain the high competence scores obtained for the caregiver role item.(21)

The care areas in which the participants scored the lowest mean self-perceived competence values (i.e., in which they perceived themselves to be incompetent) were: family income, residential building, water supply, and household pets. Family income was the area of care in which participants felt they had the lowest level of competence. This topic can be seen as a source of discomfort, although the use of strategies to reduce inequalities in health outcomes and improve the security and financial stability of low-income families is within the scope of PHC nurses’ practice.(22) The item “residential building”, which provides shelter and protection to the family, includes, among others, aspects related to architectural barriers to health and household hygiene. The average score for this item ranged from 3.30 to 3.86. Assessment of architectural barriers allows the development of a plan to create security benefits. In addition, mastery of household hygiene, a domain related to environmental health indicators, promotes conditions conducive to the health of family members and, thus, prevents accidental crises due to the impacts of disease on family functioning.

From the point of view of developmental and functional processes, the care areas of marital satisfaction and family process had maximum scores of 3.79 to 4.02, respectively, placing these items at the low end of self-perceived competence. Interventions with couples can help strengthen their relationship, preserving the health-including the sexual health-of both elements of the marital subsystem.(23,24) The overall mean self-perceived competence scores related to the family process item are consistent with the results obtained in previous research,(14) insofar as mean values for the nurses’ self-perception of competence decreased over the course of the nursing process, culminating in the lowest mean scores at the evaluation of interventions stage. In previous studies that used the nursing process methodology in the hospital environment, the greatest challenge in implementing this methodology was also the stage of evaluating interventions.(25)

The nursing process is a dynamic and systematized methodology that encourages critical thinking. Use of this methodology will increase in-depth knowledge of the family, the formulation of nursing diagnoses, and the effectiveness of interventions. Despite the geographic limitation of this study, it provides new knowledge regarding nurses’ self-perception of their competence in the context of family assessment and intervention, demonstrating both low average scores overall and low average item scores at each stage of the nursing process. This study was designed to provide new information by combining self-perception of competence with the care area domains of the MDAIF,(6) a model of family assessment and intervention at each stage of the nursing process.

## Conclusions

The conclusions of this study refer to crucial aspects regarding nurses’ perception of their own competence in family assessment and intervention. For all areas related to family assessment and intervention and at all stages of the nursing process, the participants endorsed a low level of self-perceived competence, even in those areas in which they rated themselves as competent-namely, parental role, adaptation to pregnancy, and family planning in the development dimension of the MDAIF and caregiver role in the functional dimension of the model. In the remaining care areas of the family assessment and intervention model-family income, residential building, safety precautions, water supply (structural dimension); marital satisfaction (development dimension); and family process (functional dimension)-nurses rated themselves as lacking competence. The steady decline in self-perceived competence in the areas related to family assessment and intervention over the course of the nursing process (needs assessment, diagnosis, planning of interventions, implementation of interventions, and evaluation of interventions) was also remarkable.

One limitation of this study was geographical, as the sample was drawn solely from the northern region of Portugal, which hinders generalization and external validation of our results and creates a need for replication research in other areas of the country.

It is important to train nurses in all MDAIF care areas within their practice settings, thus facilitating awareness of the skills needed to provide the best care to families. With regard to formal learning, institutes of nursing education must include objectives and content in their curricula that enable conceptualization of the family as an open system, with evolutionary and contextual dimensions that give it an unique identity that emerges from the reciprocity of mutual interactions between its members and the environment. The implications for practice of the development of competences based on a theoretical framework should demonstrate, in future research, changes in the self-perceived competence of nurses, as well as adequate responses to the health needs of families that are amenable to nursing care.
